# Negative selection of chronic lymphocytic leukaemia cells using a bifunctional rosette-based antibody cocktail

**DOI:** 10.1186/1472-6750-8-6

**Published:** 2008-01-29

**Authors:** Salim Essakali, Dennis Carney, David Westerman, Peter Gambell, John F Seymour, Alexander Dobrovic

**Affiliations:** 1 Department of Pathology, Peter MacCallum Cancer Centre, St Andrews Place, Melbourne, Victoria 3002, Australia; 2 Universitaetsklinik Duesseldorf, Molekulare Pathologie, Moorenstr. 5, 40215 Duesseldorf, Germany; 3 Department of Pathology, University of Melbourne, Parkville, Victoria 3010, Australia

## Abstract

**Background:**

High purity of tumour samples is a necessity for accurate genetic and expression analysis and is usually achieved by positive selection in chronic lymphocytic leukaemia (CLL).

**Results:**

We adapted a bifunctional rosette-based antibody cocktail for negative selection of B-cells for isolating CLL cells from peripheral blood (PB). PB samples from CLL patients were split into aliquots. One aliquot of each sample was enriched by density gradient centrifugation (DGC), while the other aliquot of each sample was incubated with an antibody cocktail for B-cell enrichment prior to DGC (RS+DGC). The purity of CLL cells after DGC averaged 74.1% (range: 15.9 – 97.4%). Using RS+DGC, the purity averaged 93.8% (range: 80.4 – 99.4%) with 23 of 29 (79%) samples showing CLL purities above 90%. RNA extracted from enriched CLL cells was of appropriately high quality for microarray analysis.

**Conclusion:**

This study confirms the use of a bifunctional rosette-based antibody cocktail as an effective method for the purification of CLL cells from peripheral blood.

## Background

Enrichment of tumour cells to a purity of more than 90% is highly desirable for accurate results in many applications, especially for RT-PCR and microarray based expression analysis [1,2]. In B-cell chronic lymphocytic leukaemia (CLL), such purities have usually been achieved by density gradient centrifugation (DGC) and subsequent fluorescent-activated cell sorting (FACS) or by magnetic cell sorting (MCS) for CD19 positive cells [1].

Studies focusing on expression analysis in CLL utilising microarrays report median purities of 88 and 90% of CD19 positive cells using DGC [3,4] though it is likely that selection occurred for samples with high purity. One study applying DGC and FACS of mononuclear cells reported purities of between 90 and 95% of CD5–CD19 co-expressing cells [5]. Three studies [6-8] reported purities higher than 97% of CD19 positive cells after DGC and MCS. Although high purity is achieved with FACS and MCS, both are time and cost intensive procedures which often are limited in terms of tumour cell yields and applicability, since they require expensive equipment and the processing time depends on the sample volume. Another potential disadvantage is that they are positive selection approaches which might alter gene expression through the activation of cell surface receptors [1].

Our study focused on adapting a negative selection method that could offer the required purity after the DGC step thereby markedly cutting down the time and cost of sample processing and reducing the risk of altering the gene expression pattern.

We used a bifunctional antibody cocktail for B-cell enrichment (RosetteSep™ (RS)) that binds erythrocytes (via glycophorin) on one side and white cell populations other than B-cells (via the CD2, CD3, CD16, CD36, CD56 and/or CD66b antigens) on the other side thus forming dense rosettes of erythrocytes surrounding the unwanted white blood cells when added to whole blood. The increased density of the rosetted cells results in their pelleting by subsequent DGC. This combination of RS incubation and subsequent density gradient centrifugation (RS+DGC) thus results in the depletion of undesired cells and leaves purified B-cells behind that can be harvested from the interface [9]. Here, we investigate whether RS+DGC can also effectively isolate CLL cells at high purity from peripheral blood (PB).

## Results and Discussion

A preliminary experiment was used to assess the optimal RS concentration that resulted in the best purity. Aliquots of three CLL samples were treated with 50, 60, 70 and 80 μl RS/ml PB to monitor the effect on the resulting purity. The experiments indicated that a concentration of 70 μl RS/ml PB resulted in the best purity (see Additional File 1). This was the concentration used to subsequently enrich all CLL samples.

**Figure 1:**
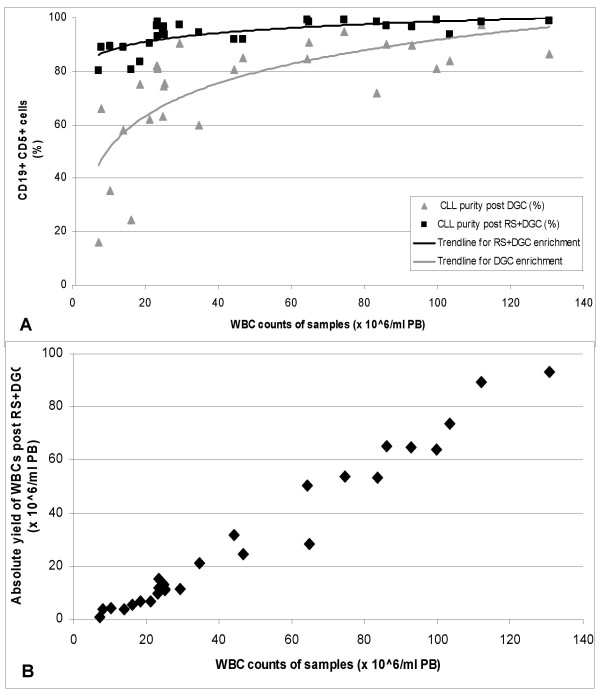
purity of CD5+ CD19+ cells in the same sample after DGC or RS+DGC enrichment (A) and absolute yield of WBCs after RS+DGC enrichment (B) plotted against the respective white blood cell count.

Enrichment with RS+DGC was performed in less than 90 minutes and showed higher purities of CD5/CD19 co-expressing cells for every sample compared to the enrichment with DGC alone. The analysed PB samples of CLL patients showed an average CLL cell purity of 74.1% (ranging from 15.9 to 97.4%) after DGC (see Figure 1A and Additional Files 2 and 3). After RS+DGC enrichment, the same samples exhibited an average CLL cell purity of 93.8% (ranging from 80.4 to 99.4%). The average purity of CD5/CD19 co-expressing cells was raised from 74.1% after DGC to 93.8% after RS+DGC. The average percentage of CD5^- ^CD19^+ ^(normal B-cells), CD5^- ^CD19^- ^(natural killer cells and monocytes) and CD5^+ ^CD19^- ^cells (T-cells) was reduced from 1.4, 10.1 and 14.4 to 1.0, 3.5 and 1.6% respectively after RS+DGC (see Additional File 3).

The purity of the enriched CLL cells (based on CD5/CD19 co-expression) increased with the WBC count of the samples (see Figure 1A). RS+DGC enrichment resulted in a CLL purity of greater than 90% for all 23 of the 29 samples that showed a WBC count higher than 20 × 10^6^cells/ml PB, while the 6 samples with a WBC count between 7 and 20 × 10^6^cells/ml PB showed CLL purity between 80 and 90% after RS+DGC enrichment. The consistently higher purities achieved with RS+DGC in all 29 samples compared to DGC alone show the efficacy of the rosette based enrichment method, and is comparable with purities achieved by MCS and FACS and superior in terms of time and cost (see Table 1).

**Table 1:** Comparison of the discussed four different enrichment approaches

	**DGC**	**RS+DGC**	**DGC+MCS**	**DGC+FACS**
**Purity**	ca. 70% ^a^	ca. 93% ^a^	> 98% ^d^	90 – 95% ^f^
	CD5^+^/CD19^+^	CD5^+^/CD19^+^	> 93% ^e^	CD5^+^/CD19^+^
			CD19^+^	
	86% (± 14%) ^b^			
	CD19^+^			
	90% (± 7%) ^c^			
	CD5^+^/CD19^+^/CD23^+^			
**Time of procedure**	< 60 mins.	< 90 mins.	> 120 mins (increasing with sample volume)	> 120 mins (increasing with sample volume)
**Selection method**	negative	negative	positive	positive
**Limitations**	highly depending on WBC count	dependent on WBC count when < 20 × 10^6 ^cells/ml PB	long process time and expensive	long process time and very expensive
**cost**	low	medium	medium-high	high
**Required Infra-structure**	centrifuge	centrifuge	centrifuge + MCS Sorter	centrifuge + FACS sorter

DGC: density gradient centrifugation, RS+DGC: RosetteSep incubation prior to DGC, DGC+MCS: DGC and subsequent magnetic cell sorting, DGC+FACS: DGC and subsequent fluorescent activated cell sorting.

^a ^average purity experienced in our study

^b ^purity reported by Haslinger et al., 2004

^c ^purity reported by Falt et al., 2005

^d ^purity reported by Rosenwald et al., 2004 and Wiestner et al., 2003

^e ^Huttman et al., 2006

^f ^purity reported by Stankovic et al., 2004

Not surprisingly, the cell yield also depended on the WBC count (see Figure 1B and Additional File 4). The number of cells harvested can be regulated by increasing (or decreasing) the volume of blood to be processed and by adjusting the volume of added RS antibody cocktail accordingly without any effect on the processing time of 90 minutes. This is another potential advantage over FACS and MCS where increased cell numbers require increased processing time.

The RNA extracted from enriched cells using RS+DGC displayed an average RIN of 8.9 (ranging from 7.7 to 9.5), indicating high-quality RNA (see Additional File 3) that subsequently gave excellent results on microarray analysis (data not shown).

## Conclusion

This study shows that negative selection using a bifunctional rosette-based antibody cocktail is an effective method to isolate CLL cells of high purity, especially in samples with a WBC count above 20 × 10^6 ^cells/ml. The short purification time, the independence from expensive and time consuming procedures, such as FACS and MCS (see Table 1), and the flexible adjustment of cell yields makes RS+DGC an attractive purification method for a wide spectrum of downstream applications, particularly expression analysis utilising microarrays, in which a CLL purity of >90% is desirable.

## Methods

Peripheral blood (PB) samples of CLL patients were obtained as part of a study approved by the Peter MacCallum Ethics of Human Research Committee. The white blood cell (WBC) count ranged from 7.81 to 437.08 × 10^6^/ml and averaged 76.1 × 10^6^/ml. The diagnosis of CLL was based on earlier examination of the patients' blood film and immunophenotyping for CD3, CD4, CD5, CD8, CD10, CD16, CD19, CD20, CD22, CD23, CD38, CD45, CD56, FMC7 and surface immunoglobulin light chain expression.

Blood from CLL patients was incubated with RosetteSep™ (StemCell Technologies Inc., Vancouver, British Columbia, Canada) (RS) at a concentration of 70 μl/ml PB in the dark at room temperature for 20 minutes with gentle manual swirling every 5 minutes. As a control, an aliquot of the same sample was processed the same way except without addition of RS. After incubation, both aliquots of blood were diluted with 4 volumes (rather than 2 volumes as recommended by the manufacturer) of Dulbecco's phosphate buffered saline (PBS) containing 2% foetal bovine serum as otherwise we found that blood samples with high WBC counts were insufficiently diluted for efficient separation. The samples were then underlaid with 3 ml Lymphocyte Separation Medium (MP Biomedicals, Aurora, OH) and centrifuged for 20 minutes at 1,200 g. The enriched cells were subsequently harvested from the interface and washed once in 2 volumes Dulbecco's PBS with 2% foetal bovine serum by centrifuging for 10 minutes at 200 g.

The purity of the enriched cell population was analysed by staining with a panel of fluorescently labelled antibodies (BD Biosciences, San Jose, CA) in three different tubes. (Tube 1: CD5-FITC, CD10-PE, CD19-PerCP and CD45-APC. Tube 2: FMC7-FITC, CD23-PE, CD19-PerCP and CD45-APC. Tube 3: CD22-FITC, CD38-PE, CD20-PerCP and CD45-APC). Data acquisition was carried out using a FACScalibur-cytometer (BD Biosciences) and Cell Quest software (BD Biosciences). Ungated data analysis was conducted using Cytomics RXP software (Beckman Coulter, Fullerton, CA).

The diagnosis of CLL was reconfirmed by assessing positivity for CD5, CD19, CD23 and CD45, weak positivity for CD20; weak positivity or negativity for CD22 and FMC7 and negativity for CD10. After confirming the CLL diagnosis, the CLL purity was assessed by the co-expression of CD5 and CD19.

RNA was extracted from RS+DGC enriched CLL cells by applying a combination of the Trizol-protocol (Invitrogen, Carlsbad, CA, USA) and the RNeasy Micro Kit (Qiagen, Hilden, Germany). The RNA Integrity Number (RIN) of the extracted RNA was determined using the 2100 Bioanalyzer (Agilent, Santa Clara, CA) according to the manufacturer's protocol.

## Abbreviations

CLL Chronic Lymphocytic Leukaemia

DGC density gradient centrifugation

FACS fluorescent-activated cell sorting

MCS magnetic cell sorting

PB peripheral blood

PBS Dulbecco's phosphate buffered saline

PCR polymerase chain reaction

RIN RNA Integrity Number

RS RosetteSep™

RS+DGC addition of RosetteSep B-cell enrichment cocktail prior to density gradient centrifugation

RT-PCR reverse transcription polymerase chain reaction

WBC white blood cell

## Authors' contributions

SE adapted the methodology, carried out the laboratory work, participated in experimental design, and drafted the manuscript. DC participated in the experimental design and supplied clinical specimens. DW participated in the experimental design and supplied clinical specimens. PG assisted with the immunophenotyping. JFS supplied clinical specimens. AD participated in experimental design, supervised the laboratory work and brought the manuscript to its final form. All authors read and approved the final manuscript.

## Supplementary Material

Additional file 1 CD5/CD19 immunophenotyping of sample CLL22 (1), CLL35 (2) and CLL36 (3) after RS+DGC enrichment using either 50 μl (A), 60 μl (B), 70 μl (C) or 80 μl (D) RosetteSep/ml whole blood. Data indicates that a concentration of 70 μl RosetteSep/ml whole blood gives the highest purities of CD5^+ ^CD19^+ ^cells. Click here for file

Additional file 2 CD5/CD19 immunophenotyping of sample CLL2 after DGC (left) and after RS+DGC enrichment (right). Figure shows the CD5/CD19 cell surface expression of one sample (CLL2) post DGC, and post RS+DGC enrichment determined by immunophenotyping. The RS+DGC enrichment lead to a significant increase in the proportion of the CD5^+ ^CD19^+ ^cells. Click here for file

Additional file 3 White Blood Cell (WBC) counts of fresh CLL peripheral blood samples and their examination for CLL purity after density gradient centrifugation DGC and RosetteSep incubation prior to DGC (RS+DGC) enrichment. The data is sorted by ascending WBC count. RNA Integrity Numbers (RIN) for RNA extracted from CLL cells after RS+DGC enrichment are shown. The figure shows the WBC counts of all CLL peripheral blood samples and the respective purity of the CD5^- ^CD19^+^, CD5^+ ^CD19^+^, CD5^- ^CD19^- ^and CD5^+ ^CD19^- ^fractions after DGC and after RS+DGC. This table also displays RNA Integrity Numbers for RNA extracted from purified CLL cells using RS+DGC. Click here for file

Additional file 4 White blood cell (WBC) counts of fresh CLL peripheral blood (PB) samples and yields and purities after RosetteSep incubation prior to density gradient centrifugation (RS+DGC) enrichment. The data is sorted by ascending WBC count. The figure shows the WBC counts of all CLL peripheral blood samples and the respective cell yield and purity of the CD5^+ ^CD19^+ ^fractions after DGC and after RS+DGC. Click here for file
