# Supplementary figures and images for: Negative selection of chronic lymphocytic leukaemia cells using a bifunctional rosette-based antibody cocktail
Source: BMC Biotechnol. 2008 Jan 29;8:6. doi: 10.1186/1472-6750-8-6 (PMC2254389; doi:10.1186/1472-6750-8-6)

## Slide 1
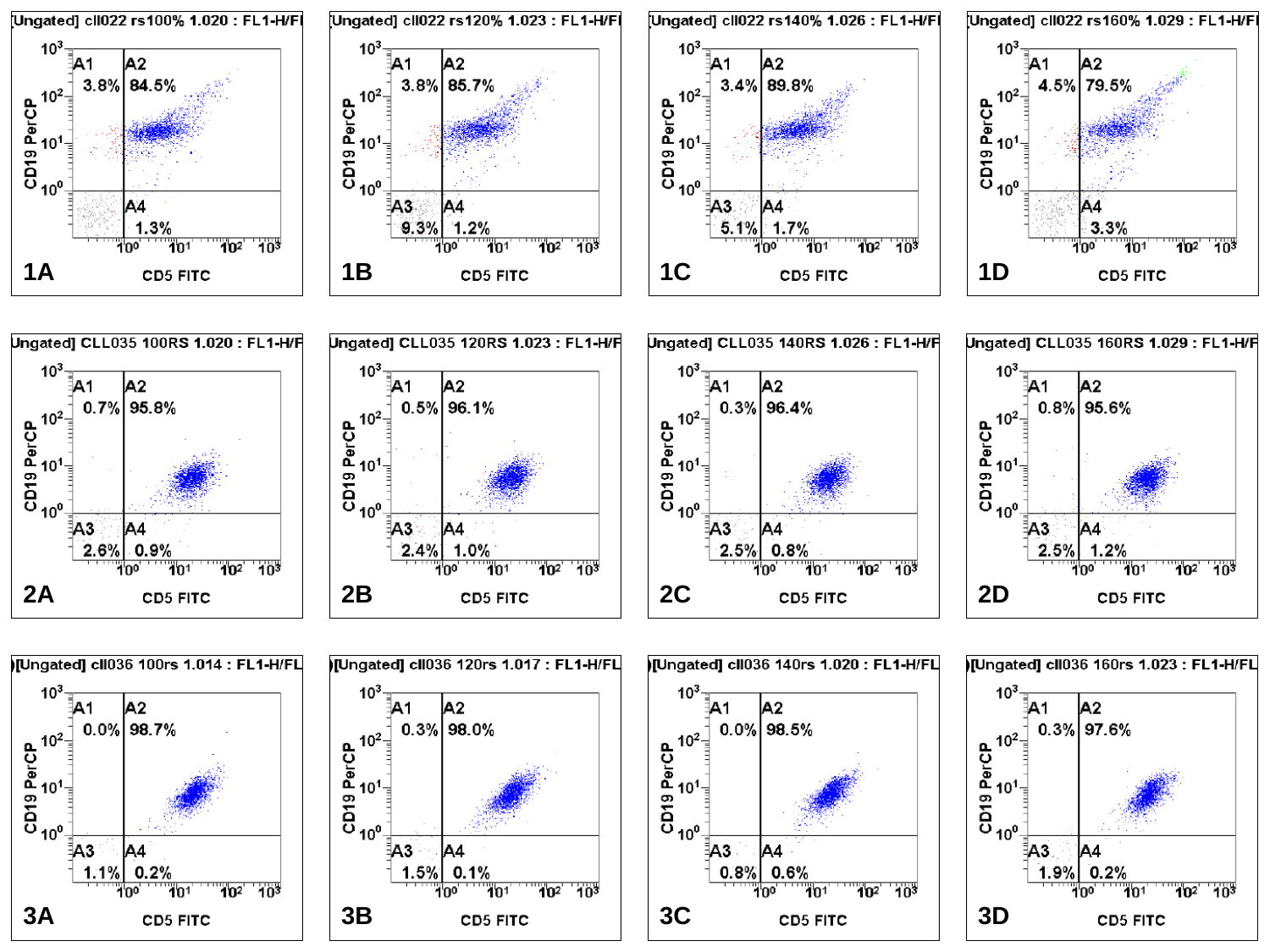

1A
1B
1C
1D
2A
2B
2C
2D
3A
3B
3C
3D

Supplement: Additional file 1 — CD5/CD19 immunophenotyping of sample CLL22 (1), CLL35 (2) and CLL36 (3) after RS+DGC enrichment using either 50 μl (A), 60 μl (B), 70 μl (C) or 80 μl (D) RosetteSep/ml whole blood. Data indicates that a concentration of 70 μl RosetteSep/ml whole blood gives the highest purities of CD5+ CD19+ cells. [file 1472-6750-8-6-S1.ppt]

## Slide 1
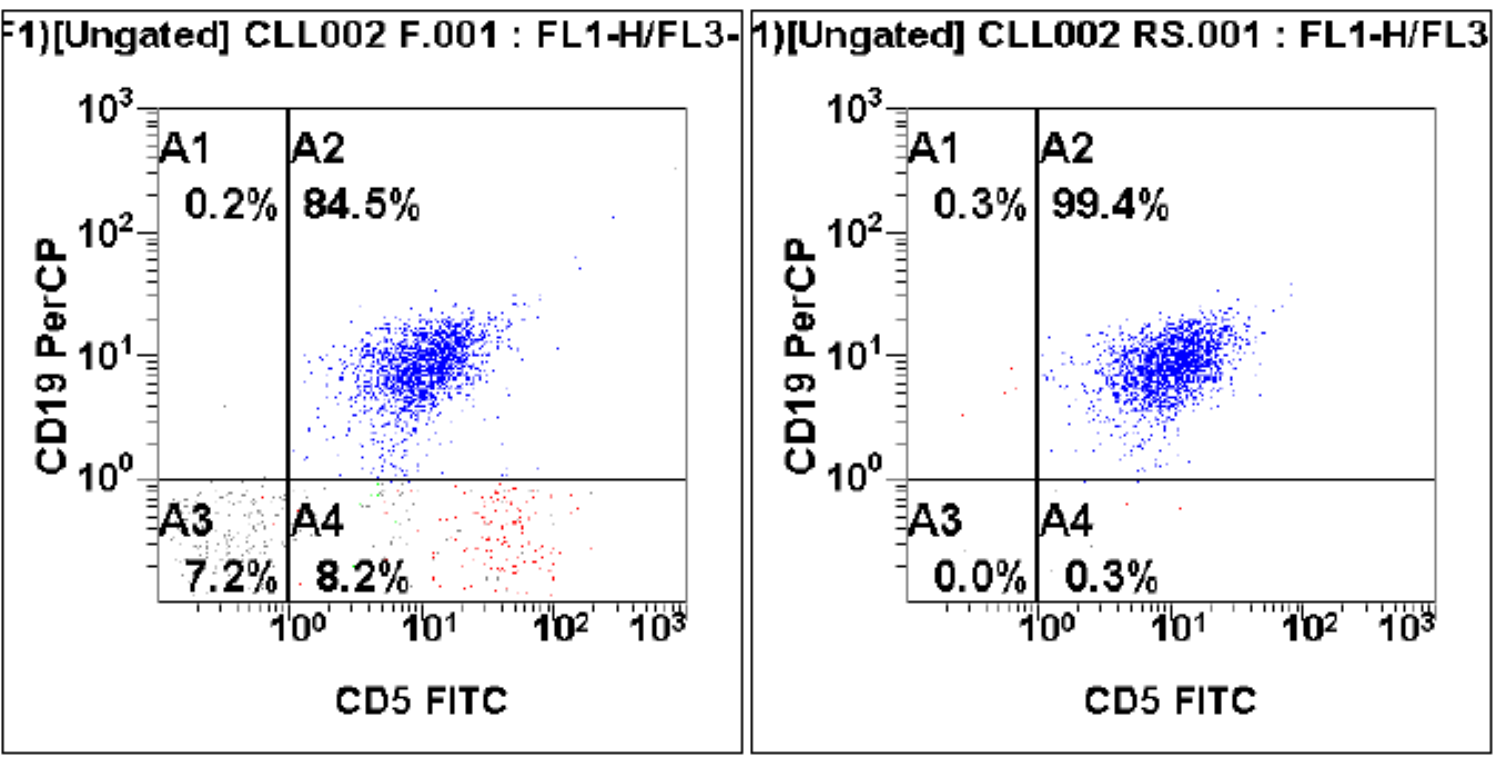

Supplement: Additional file 2 — CD5/CD19 immunophenotyping of sample CLL2 after DGC (left) and after RS+DGC enrichment (right). Figure shows the CD5/CD19 cell surface expression of one sample (CLL2) post DGC, and post RS+DGC enrichment determined by immunophenotyping. The RS+DGC enrichment lead to a significant increase in the proportion of the CD5+ CD19+ cells. [file 1472-6750-8-6-S2.ppt]
