# Supplementary material for: Negative selection of chronic lymphocytic leukaemia cells using a bifunctional rosette-based antibody cocktail
Source: BMC Biotechnol. 2008 Jan 29;8:6. doi: 10.1186/1472-6750-8-6 (PMC2254389; doi:10.1186/1472-6750-8-6)
Supplement: Additional file 3 — White Blood Cell (WBC) counts of fresh CLL peripheral blood samples and their examination for CLL purity after density gradient centrifugation DGC and RosetteSep incubation prior to DGC (RS+DGC) enrichment. The data is sorted by ascending WBC count. RNA Integrity Numbers (RIN) for RNA extracted from CLL cells after RS+DGC enrichment are shown. The figure shows the WBC counts of all CLL peripheral blood samples and the respective purity of the CD5- CD19+, CD5+ CD19+, CD5- CD19- and CD5+ CD19- fractions after DGC and after RS+DGC. This table also displays RNA Integrity Numbers for RNA extracted from purified CLL cells using RS+DGC. [file 1472-6750-8-6-S3.pdf]

Additional Material Table 1: White Blood Cell (WBC) counts of fresh CLL peripheral blood samples and their examination for CLL purity after density gradient centrifugation (DGC) and RosetteSep incubation prior to DGC (RS+DGC) enrichment. Data sorted by ascending WBC count. RNA Integrity Numbers (RIN) for RNA extracted from CLL cells after RS+DGC enrichment.

		Cells post DGC (%)				Cells post RS+DGC (%)				
sample	WBC	$CD5^-$ $CD19^+$	$CD5^+$ $CD19^+$	$CD5^-$ $CD19^-$	$CD5^+$ $CD19^-$	$CD5^-$ $CD19^+$	$CD5^+$ $CD19^+$	$CD5^-$ $CD19^-$	$CD5^+$ $CD19^-$	RIN
**CLL035**	7.2	0.9	15.9	28.3	55.0	3.7	80.4	13.5	2.3	8.8
**CLL034**	8.0	0.3	66.0	27.4	6.2	0.0	89.1	9.8	1.1	-
**CLL042**	10.3	1.2	35.5	24.3	39.0	3.3	89.3	5.2	2.2	9.0
**CLL022**	14.1	3.4	58.2	8.7	29.7	2.6	89.1	7.0	1.2	-
**CLL014**	16.1	0.1	24.3	15.1	60.5	0.0	80.5	8.1	11.0	-
**CLL013**	21.3	2.9	61.9	13.3	21.8	5.9	90.5	2.3	1.4	-
**CLL029**	23.1	1.0	82.1	6.9	10.1	0.3	97.0	1.7	0.2	8.9
**CLL031**	23.4	1.1	81.6	9.8	7.5	0.1	98.6	0.9	0.4	7.7
**CLL020**	23.4	1.7	81.2	5.8	11.2	3.0	93.0	3.8	0.2	-
**CLL016**	24.8	1.8	63.3	15.3	19.6	1.6	94.9	1.9	1.6	9.0
**CLL012**	25.1	1.2	74.4	6.8	17.6	1.1	96.8	1.2	1.0	9.4
**CLL033**	25.2	0.7	75.5	13.7	10.1	0.5	93.9	5.1	0.5	-
**CLL019**	29.4	0.2	90.6	5.3	3.9	0.0	97.5	1.9	0.6	9.3
**CLL025**	34.7	9.7	59.7	23.6	7.0	1.4	94.6	3.3	0.7	9.5
**CLL030**	44.3	0.0	80.8	5.6	13.6	0.8	91.9	6.5	0.7	9.1
**CLL028**	46.7	1.1	85.2	4.2	9.6	1.7	91.8	5.7	0.8	9.1
**CLL017**	64.3	0.2	84.5	7.2	8.2	0.3	99.4	0.0	0.3	9.0
**CLL039**	64.9	0.0	90.7	0.7	8.6	0.2	98.4	0.7	0.6	9.1
**CLL024**	74.5	0.2	95.0	2.7	2.2	0.0	99.1	0.7	0.2	9.3
**CLL021**	83.5	2.3	72.0	6.3	19.3	0.1	98.6	0.4	0.8	-
**CLL032**	86.0	0.9	90.1	6.1	2.9	0.4	96.9	2.7	0.0	8.9
**CLL002**	92.9	0.6	89.9	2.0	7.4	0.5	96.8	2.3	0.4	8.2
**CLL041**	99.8	0.0	81.1	6.7	12.2	0.0	99.1	0.6	0.3	-
**CLL001**	103.3	3.0	84.1	9.8	3.1	0.2	93.7	1.0	5.2	8.0
**CLL036**	103.3	1.0	75.0	5.6	18.5	1.4	83.7	5.7	9.2	9.1
**CLL026**	112.0	0.0	97.4	0.6	2.0	0.1	98.4	0.8	0.7	9.1
**CLL040**	130.7	0.5	86.5	11.1	1.9	0.0	98.8	1.1	0.0	8.7
**CLL023**	378.7	3.1	83.6	10.4	2.9	0.5	95.8	3.2	0.6	-
**CLL011**	437.1	1.3	83.9	9.8	4.9	0.2	92.7	4.6	2.5	-
average	**76.1**	**1.4**	**74.1**	**10.1**	**14.4**	**1.0**	**93.8**	**3.5**	**1.6**	**8.9**
