# Supplementary material for: Negative selection of chronic lymphocytic leukaemia cells using a bifunctional rosette-based antibody cocktail
Source: BMC Biotechnol. 2008 Jan 29;8:6. doi: 10.1186/1472-6750-8-6 (PMC2254389; doi:10.1186/1472-6750-8-6)
Supplement: Additional file 4 — White blood cell (WBC) counts of fresh CLL peripheral blood (PB) samples and yields and purities after RosetteSep incubation prior to density gradient centrifugation (RS+DGC) enrichment. The data is sorted by ascending WBC count. The figure shows the WBC counts of all CLL peripheral blood samples and the respective cell yield and purity of the CD5+ CD19+ fractions after DGC and after RS+DGC. [file 1472-6750-8-6-S4.pdf]

Additional Material Table 2: White blood cell (WBC) counts of fresh CLL peripheral blood (PB) samples and yields and purities after RosetteSep incubation prior to density gradient centrifugation (RS+DGC) enrichment. Data sorted by ascending WBC count.

sample	WBC count (x10^6cells/ml PB)	Absolute yield of WBCs post RS+DGC (x10^6cells/ml processed PB)	CLL purity (%)	CLL yield (x10^6cells/ml processed PB)
**CLL021**	7.2	0.8	80.4	0.6
**CLL017**	8.0	4.0	89.1	3.6
**CLL022**	10.3	4.3	89.3	3.8
**CLL011**	14.1	3.7	89.1	3.3
**CLL023**	16.1	5.4	80.5	4.4
**CLL033**	18.5	6.8	83.7	5.7
**CLL041**	21.3	7.0	90.5	6.3
**CLL034**	23.1	9.7	97.0	9.4
**CLL030**	23.4	12.0	98.6	11.8
**CLL020**	23.4	15.2	93.0	14.2
**CLL035**	24.8	13.1	94.9	12.4
**CLL042**	25.1	11.5	96.8	11.1
**CLL014**	25.2	11.1	93.9	10.4
**CLL026**	29.4	11.4	97.5	11.1
**CLL025**	34.7	21.0	94.6	19.8
**CLL019**	44.3	31.6	91.9	29.0
**CLL040**	46.7	24.6	91.8	22.5
**CLL002**	64.3	50.5	99.4	50.2
**CLL031**	64.9	28.3	98.4	27.8
**CLL039**	74.5	53.8	99.1	53.3
**CLL001**	83.5	53.2	98.1	52.2
**CLL036**	86.0	65.3	96.9	63.3
**CLL029**	92.9	64.7	96.8	62.6
**CLL016**	99.8	64.0	99.1	63.4
**CLL032**	103.3	73.8	93.7	69.2
**CLL024**	112.0	89.6	98.4	88.2
**CLL028**	130.7	93.3	98.8	92.2
**CLL013**	378.7	266.3	95.8	255.1
**CLL012**	437.1	310.7	92.7	288.0
